# Sperm epigenetics and male infertility: unraveling the molecular puzzle

**DOI:** 10.1186/s40246-024-00626-4

**Published:** 2024-06-04

**Authors:** Maryam Hosseini, Anis Khalafiyan, Mohammadreza Zare, Haniye Karimzadeh, Basireh Bahrami, Behnaz Hammami, Mohammad Kazemi

**Affiliations:** 1 Department of Genetics and Molecular Biology, Faculty of Medicine, Isfahan University of Medical Sciences, Isfahan, Iran; 2 Department of Medical Genetics, School of Medicine, Tehran University of Medical Sciences, Tehran, Iran; 3 Reproductive Sciences and Sexual Health Research Center, Isfahan University of Medical Sciences, Isfahan, Iran

**Keywords:** Sperm, Epigenetics, Infertility, DNA methylation, Histone modifications, Non-coding RNAs

## Abstract

**Background:**

The prevalence of infertility among couples is estimated to range from 8 to 12%. A paradigm shift has occurred in understanding of infertility, challenging the notion that it predominantly affects women. It is now acknowledged that a significant proportion, if not the majority, of infertility cases can be attributed to male-related factors. Various elements contribute to male reproductive impairments, including aberrant sperm production caused by pituitary malfunction, testicular malignancies, aplastic germ cells, varicocele, and environmental factors.

**Main body:**

The epigenetic profile of mammalian sperm is distinctive and specialized. Various epigenetic factors regulate genes across different levels in sperm, thereby affecting its function. Changes in sperm epigenetics, potentially influenced by factors such as environmental exposures, could contribute to the development of male infertility.

**Conclusion:**

In conclusion, this review investigates the latest studies pertaining to the mechanisms of epigenetic changes that occur in sperm cells and their association with male reproductive issues.

## Introduction

According to the World Health Organization (WHO), infertility is defined as the inability to achieve pregnancy following a minimum of 12 months of consistent and unprotected sexual intercourse [1]. Reliable statistics on the global prevalence of this problem are lacking due to regional variations; however, it is generally believed that infertility affects approximately 8–12% of couples worldwide [2]. This leads to significant psychological and social problems, which in turn result in a considerable financial burden on both patients and the healthcare systems [3].

The etiology of reproductive problems can be attributed to one or both couples. Most studies estimate that male factors contribute to 30–50% of these cases [4]. Male infertility is characterized by the incapacity of a male to impregnate a fertile female for a minimum duration of one year of unprotected intercourse [5]. Several variables such as environmental factors, testicular dysfunction, and genital tract infections have the potential to influence reproductive capabilities of men [6]. In addition, various parameters pertaining to sperm, such as its morphology, motility, concentration, and genetic and epigenetic alterations, are also considered important determinants associated with this issue [2, 7]. While many research endeavors have focused on investigating the underlying genetic factors contributing to male infertility, their efforts have only been able to elucidate 15% of the total cases [8]. As a result, much information is still needed to better explain the pathophysiology of this problem.

One specific area of interest is epigenetics, which provides valuable insights into the complex origins of various disorders, offering a deeper understanding of their development [9]. The term epigenetics has a wide range of definitions that regularly prompt debate [10]. According to a commonly used definition, epigenetic processes refer to heritable modifications in gene expression that are not brought about by alterations in the DNA sequence [11]. DNA methylation, defined as the insertion of a methyl group at the 5′ position of cytosine residues inside a CpG dinucleotide, is the most researched area in epigenetic studies [12]. However, other factors, such as histone modifications, RNAs, and chromatin remodeling also provide additional possible epigenetic information [13].

In conclusion, since male infertility accounts for a sizable portion of infertility cases and sperm epigenetic changes can be considered a significant contributor to this problem, in this study, we aimed to summarize our current knowledge of human sperm epigenetics and its direct or indirect relation with male fertility.

## Sperm epigenetic alterations and male infertility

**Fig. 1:**
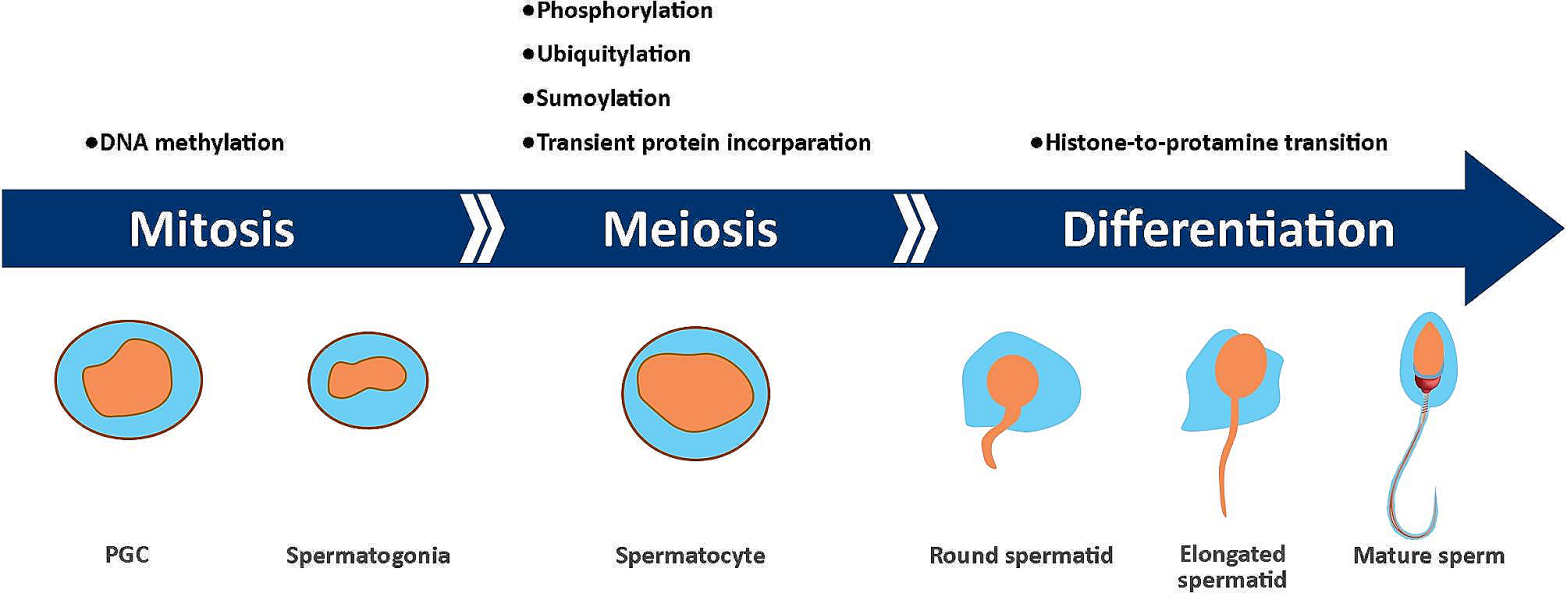
Epigenetic events during spermatogenesis. The genetic material of primordial germ cells undergoes demethylation as they migrate to the genital ridge. Global DNA methylation is established in prospermatogonia and completed before birth. Specialized histone variants are active during sperm development until the elongated spermatid stage, when they are gradually replaced by transitional proteins, preparing for transition to protamine and chromatin compaction. PGC: Primordial germ cells

**Fig. 2:**
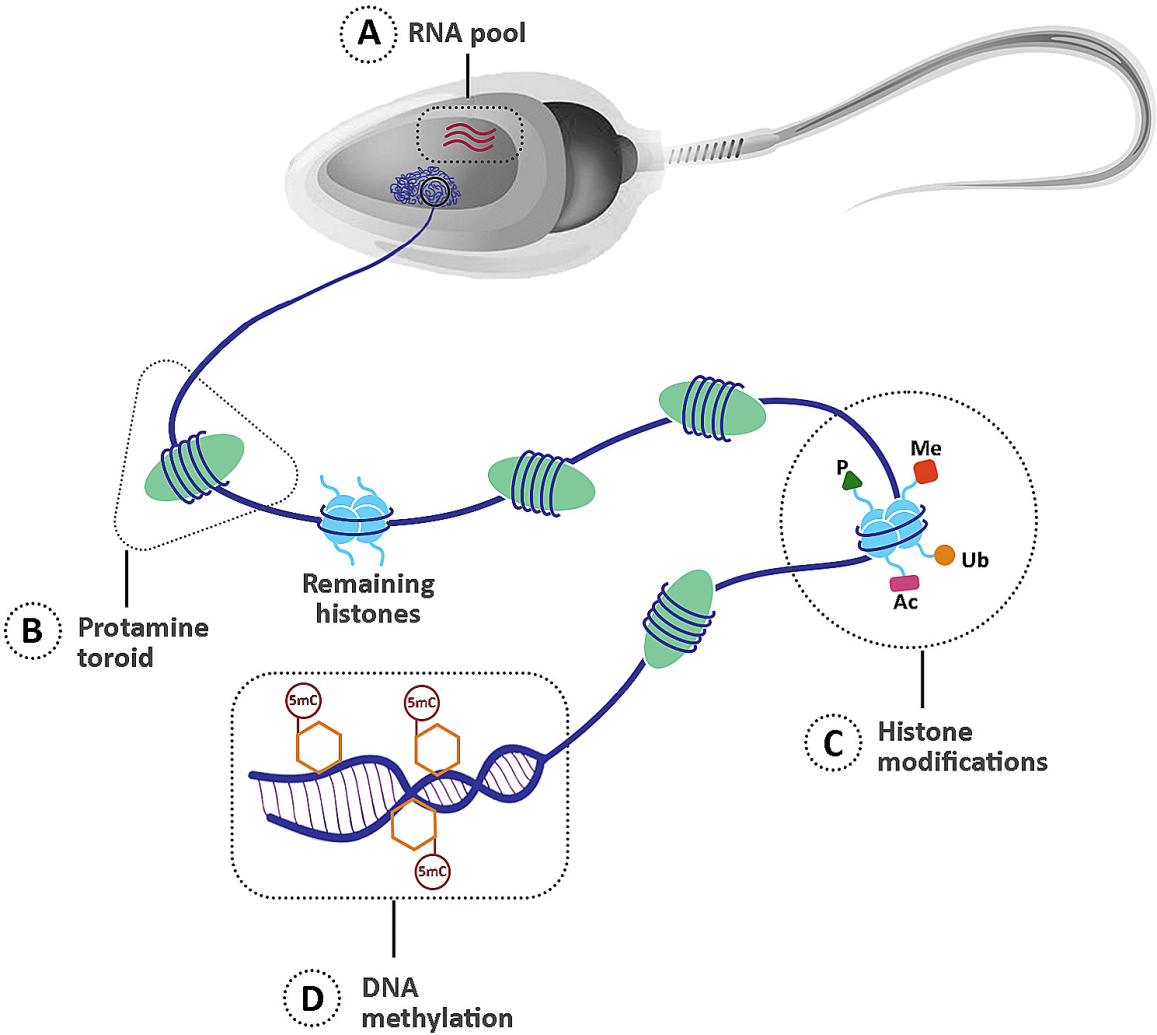
ncRNAs, histone-to-protamine transition, DNA methylation, and histone modifications are the primary epigenetic mechanisms controlling a variety of sperm activities. A complex pool of ncRNAs contain sperm-related epigenetic information. Both spermatogenesis and post-testicular maturation processes result in the acquisition of this RNA-mediated epigenetic data (**A)**. Throughout spermatogenesis, the majority of histones undergo substitution with PRMs; however, a small fraction remains in the sperm DNA **(B)**. Within sperm chromatin, a wide range of post-translational modifications take place in the remaining histones **(C)**. DNA methylation is another epigenetic marker that refers to the presence of 5mC in the DNA, which can influence the expression of genes **(D)**. ncRNAs: non-coding RNAs, PRM: Protamine, 5mC: 5-methylcytosine, Ac: Acetylation, Me: Methylation, P: Phosphorylation, Ub: Ubiquitination

During spermatogenesis, germ cell precursors transform into sperm via a sequence of controlled processes [14]. The development and differentiation of all cells, especially sperm cells, are highly dependent on epigenetic modifications. Following implantation in mammalian embryos, pluripotent cells within the epiblast differentiate into primordial germ cells (PGCs), which then undergo multiple alterations in their epigenetic characteristics [15]. The majority of these occurrences have been extensively studied using murine models, although epigenetic profiles have also been examined in sperm and embryos of other species [10]. The genome of PGCs undergoes demethylation before birth while migrating to colonize the genital ridge. In the subsequent stages, male germ cells experience an increase of up to 50% in their overall methylation levels and are nearly fully methylated at the time of birth [16]. Throughout meiosis, post-translational modifications such as phosphorylation, ubiquitylation, sumoylation, and repositioning of histone tail markers, such as H3K4me2/3 and H3K36me3 occur, and most of the core histones are gradually substituted by transitional proteins [17]. Additional modifications to histone tails, such as acetylation of lysine residues in histone 4, occur at later stages in elongating spermatids during spermiogenesis [18]. Ultimately, hyperacetylation facilitates the exchange of histones with protamines (Fig. 1) [19]. Disruptions in these epigenetic processes can be directly or indirectly related to aberrant sexual development and reproductive failure in men [20]. Chemical alterations in DNA molecules, such as chromatin remodeling, DNA methylation, histone post-translational modifications, and control of gene expression by non-coding RNAs (ncRNAs), comprise the molecular basis of the epigenetic information during these processes (Fig. 2) [14].

## Role of sperm DNA methylation in male infertility

DNA methylation is a well-studied epigenetic process in mammals that affects gene transcription and functionality [21]. Methylation occurs when a methyl group (-CH3) is transferred from S-adenosyl methionine to the fifth carbon of cytosine (5mC) by a group of enzymes called DNA methyltransferases (DNMTs) [22]. DNMTs are generally categorized into two groups: maintenance (DNMT1) and de novo (DNMT3A, DNMT3B, and DNMT3L) [23]. DNMT1 plays a crucial role in the preservation of methylation patterns during DNA replication, while maintenance DNMTs are important in the process of DNA methylation during embryonic development [24].

Research on methylation features related to male infertility has consistently shown an upward trend [25]. Changes in methylation levels of genes such as spermatogenic transposon silencer (MAEL) and GATA3 have been linked to impaired spermatogenesis [26, 27]. The deleted in azoospermia-like (DAZL) gene family plays a crucial role in embryonic germ cell development and differentiation [28]. Abnormal promoter methylation of this gene was observed in men with impaired spermatogenesis and decreased sperm function [29]. Studies have also shown that hypermethylation of DAZL is detectable in individuals with oligoasthenoteratozoospermia compared to those with normozoospermia [30, 31]. Similarly, elevated methylation levels of the CREM gene have been observed in oligozoospermic cases with aberrant protamination [32]. Hypermethylation of MTHFR gene has been reported in non-obstructive azoospermia and idiopathic infertile men; however, this phenomenon was not observed in males with obstructive azoospermia [33, 34]. Han et al., provided new insights into the mechanisms underlying defective spermatogenesis in mice. They identified SOX30 hypermethylation in non-obstructive azoospermia mice with impaired spermatogenesis [35]. Aberrant methylation can also affect the genes responsible for encoding TET enzymes. Semen samples obtained from oligozoospermia and asthenozoospermia individuals have exhibited decreased levels of TET1, TET2, and TET3 mRNAs [36]. Mice lacking TET1 exhibit a progressive decline in the number of spermatogonia [37].

Notably, a significant association exists between DNA methylation and factors related to sperm quality, including sperm motility, morphology, and DNA integrity [38, 39]. Houshdaran et al. (2007) conducted a study which stated that the process of DNA hypermethylation, particularly in the promoter region of genes such as PLAG1, PAX8, DIRAS3, MEST, SFN, NTF3, and HRAS, has the potential to negatively impact the motility and morphology of sperm [40]. The X-linked reproductive homeobox (RHOX) gene cluster plays a crucial role in spermatogenesis, germ cell viability, and male fertility [41]. A Recent research suggests that hypermethylation in this cluster may serve as a biomarker for idiopathic male infertility due to its association with significant abnormalities in various sperm parameters [42, 43]. Research has indicated that lower H19 methylation levels in infertile men is one of the possible factors affecting sperm concentration and movement [44, 45]. For instance, Minor et al. discovered a notable reduction in DNA methylation of the H19 gene in the testicular sperm of azoospermic men compared to fertile individuals [46]. Additionally, a significant reduction in the methylation levels of the H19 gene has been observed in the testicular sperm of azoospermic men compared to those in the fertile group [46, 47]. Beyond H19, disruptions in the methylation patterns of additional imprinted genes are also implicated in male infertility. The MEST and SNRPN genes demonstrate maternal imprinting, indicating that they undergo methylation in the oocyte but remain unmethylated in sperm [44, 45]. A recent meta-analysis on sperm DNA methylation aberrations of imprinted genes revealed considerably elevated methylation levels of these genes in idiopathic infertile men [48]. Other studies have also identified aberrant methylation of MEST in various reproductive issues in men including low sperm concentration, motility, and abnormal sperm morphology in idiopathic infertile males [49]; complete or incomplete maturation arrest of primary spermatocytes in azoospermic patients [50] and decreased testicular volume, elevated levels of follicle-stimulating hormone (FSH), and abnormal protamine (PRM) ratio in oligozoospermic cases [49, 51]. Another study on couples facing recurrent pregnancy loss reported hypermethylation of MEST gene in the sperm of male partners [52]. Oligozoospermic infertile males showed hypomethylation of GNAS and DIRAS3 imprinted genes compared to fertile individulas [53, 54]. Animal studies provide additional support for the association between male infertility and aberrant DNA methylation at the GNAS gene [55].Table 1 represents an organized list of genes whose impaired methylation can affect male fertility based on the type of sperm abnormality.

**Table 1:** List of aberrantly methylated genes and their related sperm abnormalities

Condition	Gene name	Function	Reference
**Normozoospermia**	MTHFR	Methylation regulator	[197]
	HSPA1L	Molecular chaperone	[198]
	HSPA1B		
	LINE1	Repetitive sequence	[199]
	NBL2		
	ALU YB8		
	D4Z4		
**Oligo-/astheno/ teratozoospermia**	XRCC1	Enzyme binding	[200]
	MLH1	DNA mismatch repair	[201]
	MEST	Hydrolase	[202]
	GNAS	G-protein alpha subunit	
	BCAN	Extracellular matrix formation	[203]
	CPEB2	Tumor suppressor	
	CRISPLD1	Cysteine-rich secretory protein	
	EZH2	Histone methyltranferase	
	HDAC4	Histone deacetylase	
	HLA-C HLA-DQA1 HLA-DRB6	Histocompatibility antigen Histocompatibility antigen Histocompatibility antigen	
	JMJD1C	Histone demethylase	
	KDM4C	Histone demethylase	
	LPHN3	Member of GPCR	
	SPATA7	Microtubule cytoskeleton organization	
	SPATA16	Testis-specific protein	
	SPATA22	Meiosis-specific protein	
	DLGAP2	Membrane-associated protein	
	GATA3	Transcriptional activator	
	MAGI2	Membrane-associated protein	
	TP73	Tumor protein	
	VDR	Transcription factor	[204]
	MTHFR	Methylation regulator	[205]
	RHOXF1, RHOXF2	Transcription factor	[42]
	SNRPN	Small nuclear ribonucleoprotein	[186, 205, 206]
	DAZL	Gametogenesis	[31]
	LINE1	Repetitive sequence	[207]
**Non-obstructive azoospermia**	MTHFR	Methylation regulator	[208]
	SOX30	Transcription factor	[35]
	DNMT1 DNMT3A DNMT3B	DNA-methyltransferase	[209]
	ZCCHC13	Zinc finger	[210]
	TDRD1	Repressor of transposable elements	[211]
	PIWIL2	Endoribonuclease	
**Oligozoospermia**	MTHFR	Methylation regulator	[208, 212]
	SPATA4 SPATA5 SPATA6	Apoptosis regulator ATP-dependent chaperone Myosin light chain binding	[213]
	GNAS	G-protein alpha subunit	[214]
	SNURF	SNRPN upstream reading frame	[53]

Assisted reproductive techniques (ARTs) have been used for four decades to treat infertility in couples. However, concerns have been raised about their safety [56]. Currently, there is a debate on how ART affects the imprinting and epigenetic reprogramming processes in gametes and early-stage embryos [57]. Approximately 41% of individuals undergoing ART exhibit aberrant methylation in their sperm cells [58]. Conversely, a link exists between the modified methylation patterns of imprinted genes in sperm and less promising results in assisted reproductive technology (ART) [59]. Additionally, research has demonstrated that the DNA methylation patterns displayed by sperm cells can serve as a predictive indicator of embryo quality in the context of in-vitro fertilization (IVF) [60].

## Sperm histone modifications and male infertility

Nucleosomes are fundamental units of chromatin, consisting of around 150 base pairs of DNA wrapped around a histone octamer containing two sets of H2A, H2B, H3, and H4 [61]. Histone modification is a post-translational process that induces covalent changes in lysine-enriched terminal regions of histones, with a particular emphasis on H3 and H4 proteins [62]. These alterations can have both positive and negative effects on the binding of regulatory factors to DNA, which can either promote or inhibit gene activation [63].

Acetylation of histones H3 and H4, methylation of histone H3 at lysine 4 (H3K4), and ubiquitination of histone H2B, can help activating a gene [64]. On the other hand, gene expression can be suppressed by ubiquitination of H2A and methylation of H3K9 and H3K27. Histone marks H3K4 and H3K27 may have dual roles in regulating gene expression [65].

Histone modifications are commonly observed during various stages of spermatogenesis, fertilization, and embryonic development [66]. Numerous studies have demonstrated that aberrant histone changes throughout spermatogenesis may impair male fertility and sperm production [9]. Schon and colleagues revealed a considerable distinction in histone modifications between typical and atypical sperm samples, highlighting the significance of these alterations in sperm function and reproductive potential [67].

Histone H4 hyperacetylation (Hypac-H4) enhances the interaction of transcription factors with chromatin, boosts the accumulation of bromodomain proteins, and ultimately triggers nuclear reorganization [68]. Hypac-H4 has been shown to be crucial for the production of healthy morphological sperms and histone-to-protamine transition [69]. Men experiencing spermatogenesis impairments and spermatid arrest show a significant reduction in the quantity of Hypac-H4 in their sperm [70]. Although histone acetylation is often associated with gene transcription, it is crucial to note that sperm cells are transcriptionally dormant [71]. Histone acetylation is transferred from sperm to oocyte and this is how it contributes to gene expression regulation in early embryos [72].

Histone methylation is another type of histone modifications. H3 trimethylation at lysines 4 and 36 (H3K36me3, H3K4me3) are linked to active transcription, whereas H3 trimethylation at lysines 9 and 27 (H3K9me3, H3K27me3) promotes transcriptional repression [73]. Ejaculated samples exhibiting low sperm motility display a higher abundance of H3K4me1, H3K9me2, H3K4me3, H3K79me2, and H3K36me3 markers [74]. On the other hand, Yuen et al. revealed a relationship between decreased levels of H3f3b factor (responsible for encoding histone variant H3.3), and the presence of abnormal spermatozoa in mice [62].

Histone phosphorylation is another epigenetic modification that plays a crucial role in chromatin reorganization during spermatogenesis and DNA compaction within the sperm nucleus [69, 75]. Therefore, sperm dysfunction and male infertility can be linked to defects in histone phosphorylation as well.

## Histone-to-protamine transition and male infertility

The transition from traditional histones to PRMs is a crucial stage in spermatogenesis [76]. Protamination helps to organize a huge quantity of DNA into a tiny sperm nucleus for effective nuclear transfer to the egg during fertilization [9]. It is a necessary process for sperm movement and shielding its DNA from oxidation and harmful chemicals in the female reproductive canal [77]. PRMs are arginine-rich, basic, small proteins that are extensively produced in male germ cells [78]. They are primarily categorized into three types including PRM1, PRM2, and PRM3 [79]. PRM1 is generated as a fully formed protein expressed in all mammals, whereas PRM2 is produced as a precursor and is selectively expressed in certain species, such as primates, some rodents, rabbits, hares, and horses [80, 81]. PRM3 is defined as a PRM2 isoform that is expressed in rats, humans, apes, and old world monkeys [82]. The process of chromatin condensation during spermatogenesis involves multiple steps [83]. The removal of testis-specific histones (TH2A, TH2B, H3t, H4, and H1t) involves the acetylation of their N-terminal lysine residues because this process reduces their affinity for negatively charged DNA [13, 84]. In the process of transitioning, some lysine-rich basic proteins named transition proteins 1 and 2 (TP1 and TP2) replace histones [69]. Since TP1 intercalates between DNA bases, causing its local melting, it likely supports the eviction of histones, whereas TP2 promotes DNA condensation by increasing its melting temperature [85]. Finally the highly conserved N-terminal segment of PRM2, commonly known as cleaved PRM2 (cP2), is removed while, its carboxy-terminal segment, referred to as mature PRM2 (mP2), collaborates with PRM1 to induce chromatin hypercondensation [86].

Insufficient protamination is associated the production of sperms that are more vulnerable to both endogenous and exogenous agents [82]. Ma et al. suggest that the improper replacement of histones with PRMs may be one of the contributing factors in familial teratozoospermia with amorphous-headed sperm [87]. Research has demonstrated that infertile samples have significantly lower levels of PRM2 compared to fertile ones and this can be linked to decreased sperm count, motility, membrane integrity, and abnormal morphology [88]. Schneider et al. reported that mice with heterozygous PRM2 genotypes have normal sperm shape and motility, while PRM2-/- sperms exhibit impaired histone-to-protamine exchange, disrupted DNA hypercondensation, severe membrane abnormalities, and movement issues [89]. Unlike PRM2, where the deletion of one allele does not significantly affect male fertility, the disturbance of just a single allele of PRM1 leads to reactive oxygen species (ROS)-induced DNA damage, decreased sperm motility, an imbalanced PRM1/PRM2 ratio, and subfertility [90].

Under normal circumstances, humans express PRM1 and PRM2 in almost equal amounts, resulting in a PRM1/PRM2 ratio close to one [91]. Changes in this ratio are reported in males with poor-quality sperms, DNA fragmentation (SDF), and reproductive issues [92, 93]. For instance, Amor et al. stated that in comparison to fertile males, PRM1/PRM2 is much higher in the subfertile group and it is positively associated with SDF [94]. According to numerous studies, deficiencies in mP2 or its compartments are associated with adverse fertility outcomes. Decreased levels of ROS scavenger enzymes were observed in PRM2-deficient mouse sperm. The diminished production of these proteins can lead to loss of sperm antioxidant capacity, triggering a cascade of oxidative stress-induced reactions and subsequent DNA damage. In addition, these sperms were fully immotile and exhibited reduced viability as a result of severe membrane integrity problems [95]. Even though PRM2’s cP2 domain is not a component of the mature protein, it is necessary for effective chromatin condensation. Previous research has shown that sperm from cP2-deficient mice exhibit insufficient PRM incorporation, altered PRM ratio, retention of transition proteins, ROS-mediated degradation, and complete DNA fragmentation [96]. Multiple studies have indicated to the accumulation of PRM2 precursors in infertile men. Elevated levels of PrePRM2 are associated with disrupted histone-to-protamine exchange, compromised DNA compaction, increased risk of physical and chemical damage to the genome, and poor rates of embryo implantation after intracytoplasmic sperm injection (ICSI) [97, 98].

## Sperm-borne RNA molecules

The central dogma asserts that DNA undergoes transcription to produce mRNA, which is then translated into proteins. However, empirical evidence demonstrates that protein-coding genes alone cannot fully explain the complexity of higher organisms [99]. According to recent high-throughput investigations, up to 90% of the eukaryotic genomic DNA is transcribed. Only 1–2% of these transcripts encode proteins, whereas the majority are ncRNAs [100]. ncRNAs are divided into two categories: (1) short ncRNAs (< 200 nucleotides), which include microRNAs (miRNAs), small interfering RNAs (siRNAs), Piwi-interacting RNAs (piRNAs), small nucleolar RNAs (snoRNAs), transfer RNAs (tRNAs), and small nuclear RNAs (snRNAs), and (2) long ncRNAs (lncRNAs) that are > 200 nucleotides in length [101]. Over the past few years, a complex population of sperm RNAs, including both coding and non-coding transcripts, has been identified [102]. These RNAs are thought to be involved in various functions, including fertilization, and can serve as markers of sperm quality index [103]. Emerging research has expanded the epigenetic definition to include regulatory mechanisms mediated by ncRNAs owing to their ability to modulate gene expression without altering the underlying DNA sequence [104]. However, the classification of ncRNAs as regulators of gene function within the realm of epigenetics is a subject of ongoing debate, sparking discussion regarding their proper categorization. This study does not aim to settle this matter. In this review, the broader perspective that ncRNAs play a role in epigenetic regulation is embraced, and consequently, studies linking them to male infertility are explored.

## miRNAs

**Fig. 3:**
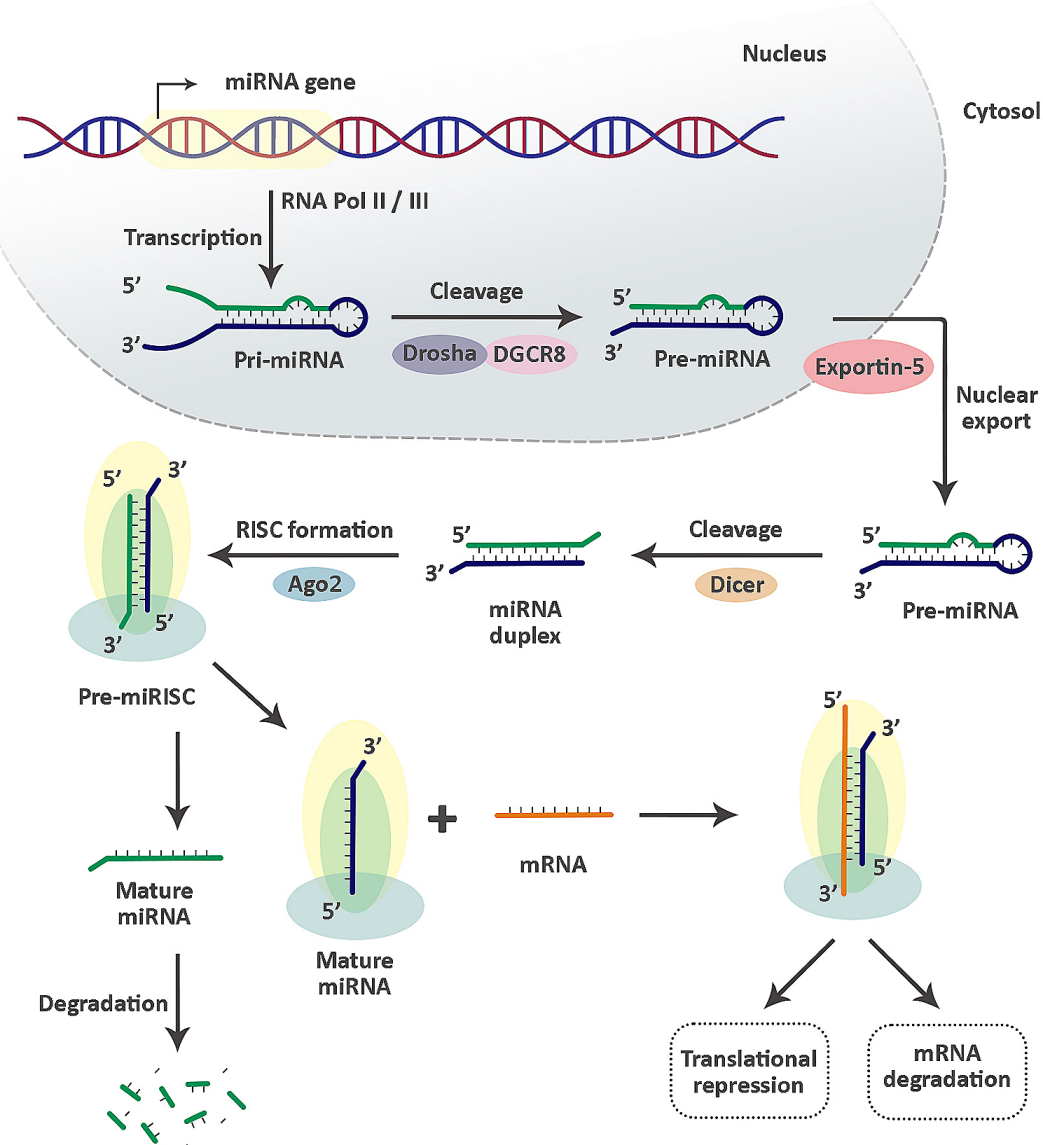
miRNAs biogenesis. Pri-miRNAs are transcribed by RNA polymerase II/III. Following transcription, a microprocessor complex comprising an RNA-binding protein, DGCR8, and a ribonuclease III enzyme called Drosha cleaves the base of the pri-miRNA hairpin, generating pre-miRNA. These are then exported to the cytoplasm by XPO5/RanGTP complex, where an RNase III endonuclease, Dicer, removes their terminal loop. Subsequently, miRNAs are integrated into the RISC complex, which includes proteins like Ago2. Ago2 plays a crucial role in either retaining or discarding one strand of the miRNA duplex. The retained guide strand is employed by the RISC for ongoing functionalities, whereas the discarded passenger strand undergoes degradation. miRNA: microRNA, Pri-miRNAs: Primary miRNAs, DGCR8: DiGeorge syndrome critical region 8, Pre-miRNA: Precursor miRNA, XPO5: exportin 5, RISC: RNA-induced silencing complex, Ago2: Argonaute RISC catalytic component 2

miRNAs are categorized as small ncRNAs, typically ranging from 19 to 25 nucleotides in length [105]. They generally target the 3′ untranslated region (3′-UTR) of transcripts to regulate their expression, although less common targeting sequences have been discovered [106]. Figure 3 provides a detailed description of their biogenesis and activity. Since their discovery, miRNAs have been studied in various cell types and tissues, and it is increasingly evident that they play a role in several biological processes [107]. miRNAs have been documented to be present in mature sperms as well as male reproductive organs such as testis, epididymis, and prostate [108]. Furthermore, it should be noted that these molecules play a crucial role in regulating sperm production at different levels [109].

It has been revealed that some miRNAs have a statistically significant negative (Let-7a, miR-7-1-3p, miR-141, miR-200a, miR-429, hsa-miR-335-5p, hsa-miR-885-5p, and hsa-miR-152-3p) and positive (miR-15b, miR-34b, miR-122, hsa-miR-629-3p, miR-26a-5p, and miR-34c-5p) connection with concentration, motility, and morphology of the sperm [110–113]. In the case of miR-26a-5p, one possible explanation is that it can suppress PTEN expression by binding to its 3’UTR, while PTEN plays an important role in sperm maturation and movement [112, 114].

Some miRNAs, including miR383 and miR122, have been found to be abnormally expressed in men with oligoasthenozoospermia [115]. Additionally, miR-19a/b-3p and miR-23a/b-3p show a negative correlation with sperm count and motility in these patients [116]. Upregulation of miR-23a/b-3p leads to downregulation of its target genes, such as PFKFB4, HMMR, SPATA6, and TEX15, which have diverse roles in spermatogenesis [117, 118].

Several miRNAs have been reported to be involved in asthenozoospermia pathology. The increased expression of miR-27a, miR-27b, and miR888-3p in the sperm of these patients suggests a possible link between these molecules and sperm motility [119, 120]. Zhou et al. discovered that miR-27b may inhibit the production of CRISP2 by targeting its 3’-UTR [119]. CRISP2 is a crucial protein in sperm that regulates its flagellar movement, acrosome response, and gamete fusion [121]. It has been revealed that in these individuals, downregulation of miR-525-3p is associated with increased SEMG1 expression [122]. SEMGs are semen key components in shaping a gelatinous meshwork with sperms trapped inside. After ejaculation, prostate-specific antigen (PSA) breaks down this meshwork, leading to sperm migration [123]. Therefore, it can be inferred that high SEMG1 expression and low miR-525-3p levels are substantially associated with poor sperm motility and morphology and, therefore, infertility [122].

Several studies have investigated the impact of miRNAs expression on varicocele associated male infertility. Ji et al. discovered that these patients have considerably lower miR-15a levels. Since this miRNA binds directly to the 3′-UTR of the stress-induced chaperone protein HSPA1B to inhibit its synthesis, it may be deduced that miR-15a plays a role in modulating cellular stress responses in sperm [124]. In another study, the downregulation of miR-21, miR-34, and miR-122a was linked to higher levels of ROS in the sperm of patients with grade III varicocele when compared to controls [125].

Estrogen has been found to have a positive effect on the ability of sperm to fertilize [126]. Estrogen receptors (ERs) are present in the male reproductive tract, germ cells, and sperm, and are crucial components of estrogen-mediated cell signaling pathways [127]. In individuals with oligospermia and reduced ERs gene expression, an increase in the levels of miR-21, miR-22, and miR-100 expression was noted [110, 128]. Therefore, it may be assumed that these miRNAs play a role male fertility due to their relationship with ER genes.

Altered expression of different sperm miRNAs is believed to affect ART outcomes and embryo development. Studies have shown that increased sperm expression of miR-99b-5p and miR-191 is positively associated with higher rates of fertilization and production of high-quality embryos during ART procedures [129, 130]. miR-34, which has been reported to be downregulated in infertile men [131], plays a role in embryonic development. Studies have demonstrated that sperms with higher levels of miR-34c-5p are more likely to develop viable embryos [132]. When sperm from male mice with miR-34b/c and miR-449 knockouts were injected into wild-type oocytes, the two-pronuclei (2PN) to zygote transition was obstructed, despite normal preimplantation events [133].

### lncRNAs

**Fig. 4:**
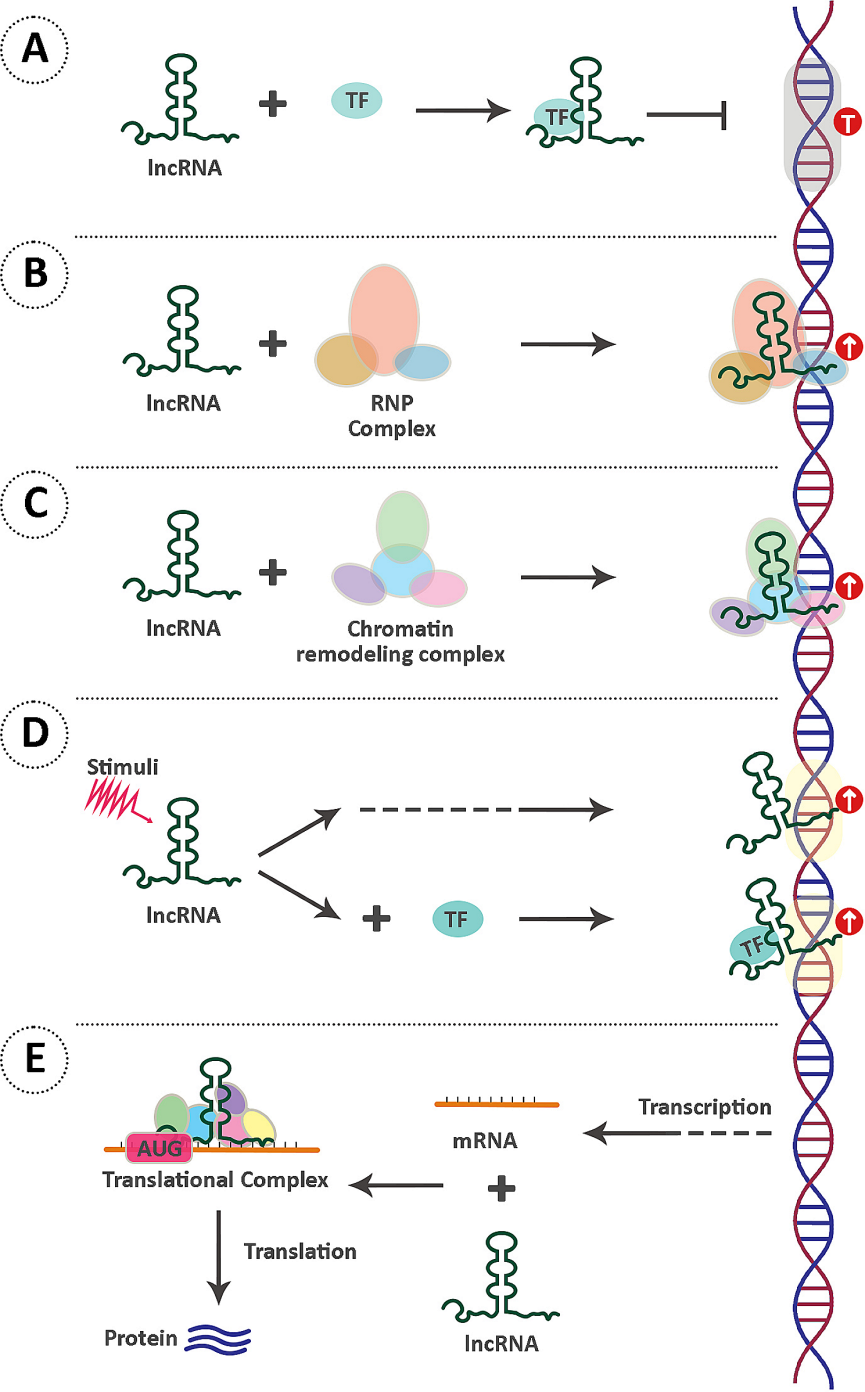
Mechanism of action of lncRNAs Decoy lncRNAs attach to miRNAs or TFs to stop them from engaging with their targets. **(A)**. Guide lncRNAs bind to their target proteins to form an RNP complex that is directed to a certain genomic locus to control gene transcription **(B)**. Scaffold lncRNAs construct scaffolding complexes in collaboration with other molecules like chromatin remodeling proteins, to regulate gene expression via epigenetic processes such as histone modification **(C)**. Signal lncRNAs regulate gene expression in response to a range of stimuli by directly binding to DNA or by forming complexes with other molecules **(D)**. SINEUP lncRNAs form translational initiation complexes that promote translation of their target mRNAs **(E)**. lncRNAs: Long non-coding RNAs, miRNAs: microRNAs, TF: Transcription factor, RNP: Ribonucleoprotein

The term “lncRNA” refers to those RNA molecules that are generally larger than 200 nucleotides in length [134]. lncRNA biosynthesis is largely similar to that of mRNAs, with a few modifications [135]. After being transcribed by RNA polymerase II, pre-mature lncRNAs undergoe 3’-polyadenylation, 5′ end capping, and alternative splicing [136]. These molecules can be categorized based on diverse features, specifically organized into five types: sense, antisense, bidirectional, intronic, and intergenic, depending on their genetic origins [137]. Their functions can be summarized into five categories, as illustrated in Fig. 4 [138]. Although numerous lncRNAs have been identified through high-throughput technologies such as next-generation sequencing (NGS), the profiling of lncRNAs in human sperm is limited [99].

Research has shown that lncRNAs are involved in the development of various sperm-related conditions. A previous study found that individuals diagnosed with oligozoospermia had abnormal expression of multiple lncRNAs, with a higher proportion being downregulated compared to upregulated ones [139]. Several lncRNAs have also been identified as important factors in asthenozoospermia and terato-asthenozoospermia. lnc32058, lnc09522, and lnc98487, were found to be significantly upregulated in the sperm of asthenozoospermic patients compared to healthy individuals. These molecules also demonstrated a significant correlation with sperm progressive motility (PR value) [140]. It has also been reported that linc00574, linc02220, and ANO1-AS2 play role in sperm motility in asthenozoospermic cases through their target genes. It is worth noting that linc00574 is transcribed from chr6q27, in close proximity to t-complex-associated-testis-expressed 3 (TCTE3) [141]. TCTE3 is an important element for flagellar structure of sperm [142], and its expression is controlled by linc00574 through a negative self-regulating mechanism. Asthenozoospermic individuals have significantly lower TCTE3 and higher linc00574 expression than healthy controls [143]. Elevation of linc02220 is associated with downregulation of DNAH5 in terato-asthenozoospermic and asthenozoospermic cases, so it can be considered a possible negative regulatory factor for DNAH5 [144]. DNAH5 is crucial for flagellum and cilia movement [145], and thus, it is associated with sperm motility and morphology. ANO1-AS2 is an antisense lncRNA located adjacent to ANO1 [146], which is a Ca2+-activated chloride channel that performs several vital functions, including ion transportation [147]. ANO1-AS2 can modulate ANO1 methylation and, consequently, alter its expression. Previous studies have shown that ANO1-AS2 and ANO1 have significant positive and negative correlations with sperm motility and morphology in individuals with terato-asthenozoospermia and asthenozoospermia [148].

Other studies have also highlighted the importance of lncRNAs in sperm concentration and structure. According to Wichman et al., male mice deficient in one of the X-linked alleles of testis-specific lncRNA-1 (Tslrn1 lncRNA) have significantly fewer sperms than controls. However, this decline had no obvious effect on mouse fertility since there are numerous lncRNAs that could compensate for Tslrn1 [149].

The lncRNA Gm2044 can be involved in sperm production through two mechanisms. First, it inhibits the translation of undifferentiated transcription factor 1 (UTF1), which is a molecular marker required to maintain a high capacity for differentiation in early spermatogonia [150]. This indirect effect reduces the ability of spermatogonia to differentiate [151]. Second, Gm2044 acts as a sponge for miR-335-3p, potentially influencing meiosis in spermatogenesis [152]. This is due to the fact that one of the targets of miR-335-3p is Synaptonemal Complex Protein 1 (Sycp1), which plays a regulatory role in meiosis [141].

Studies have suggested that both type 1 and type 2 diabetes may affect fertility [153]. Research has shown that sperm from diabetic and non-diabetic mouse models exhibit differentially expressed lncRNAs [154], indicating that some of these molecules may be associated with diabetes-related male infertility [10].

## Other ncRNAs

**Fig. 5:**
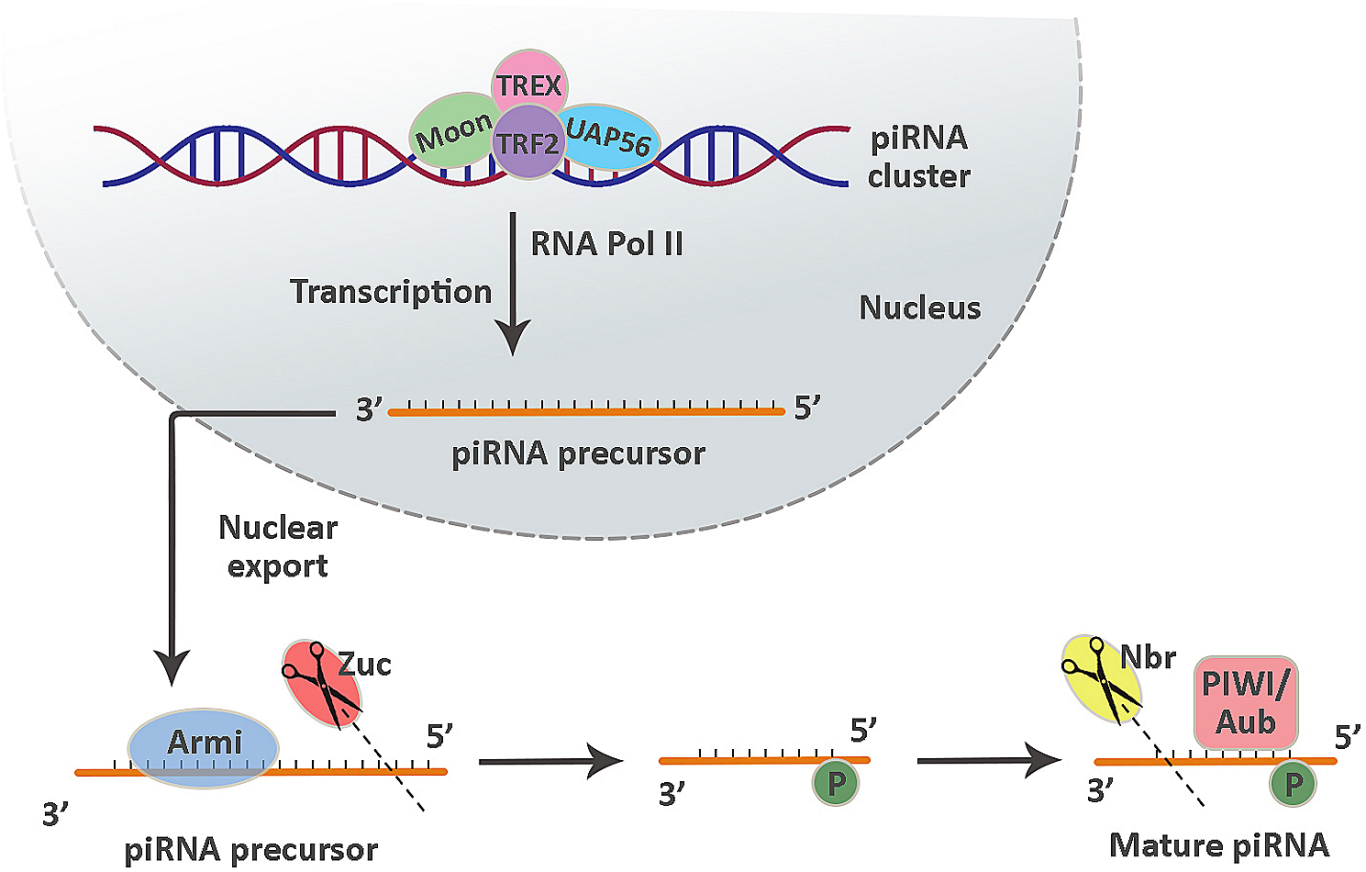
Biogenesis and main functions of piRNAs. Several factors, such as Moon, TRF2, TREX, and UAP56, participate in the generation of piRNA precursors from piRNA clusters. Subsequently, the RNA helicase Armi resolves the secondary structures present in these precursors, and then Zucchini adds a 5’ monophosphate to their structure. The maturation process of piRNAs occurs when they undergo cleavage using the 3’ to 5’ exonuclease Nibbler after being loaded onto the PIWI protein. piRNAs: Piwi-interacting RNAs, Moon: Moonshiner, TRF2: TATA-box binding protein (TBP)-related factor 2, TREX: Three prime repair exonuclease, UAP56: 56-kDa U2AF-associated protein, Armi: Armitage, Zuc: Zucchini, Nbr: Nibbler, Aub: Aubergine

Two decades ago, piRNAs were identified in the testes of Drosophila melanogaster. In contrast to miRNAs, piRNAs originate from single-stranded RNA molecules [155] and are abundant in both sperm and spermatocytes within mammalian germlines [156]. According to several lines of evidence, piRNAs participate in the identification, confrontation, and consolidation of the germline genome during fertilization [157]. piRNAs may function to safeguard genomic integrity during the early stages of embryonic development by binding to DNA and blocking the activity of repetitive and transposable elements. It is important to note that piRNAs also play a crucial role in protecting the genome during significant demethylation and remethylation events [158]. The exact process of piRNA generation remains unclear, as illustrated in Fig. 5 [159].

piRNAs are shown to be important factors in the sperm of infertile men. According to Cui et al., males with low sperm counts have downregulated levels of piR-31,704 and piR-39,888 in their sperms. As defective spermatogenesis is associated with reduced sperm counts, they proposed that piR-31,704 and piR-39,888 may be crucial for spermatogenesis. They also indicated that these two piRNAs, in conjunction with piR-40,349, were significantly upregulated in subjects exhibiting greater rates of 2PN levels after ICSI procedure [160].

**Fig. 6:**
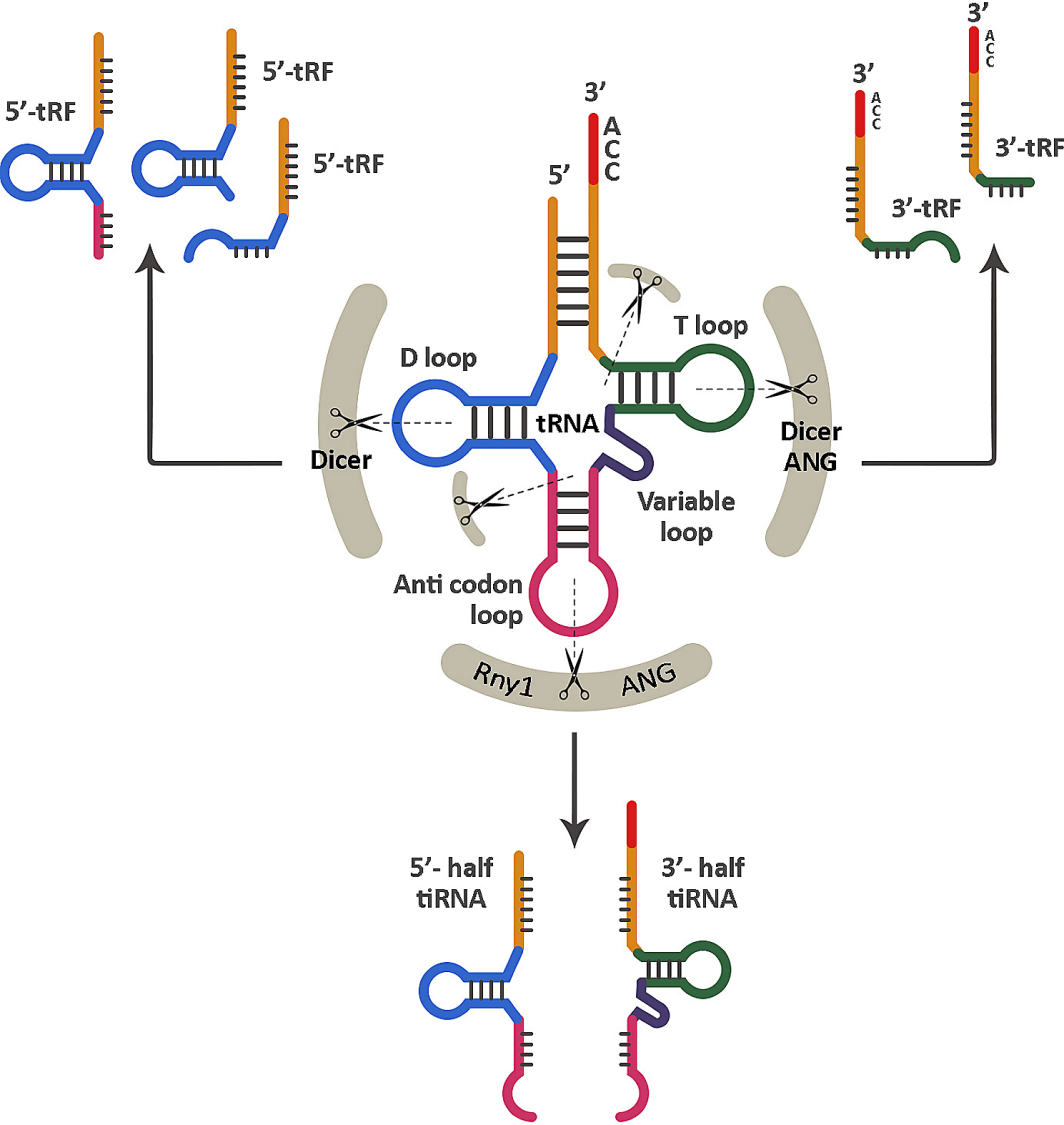
Biogenesis and main functions of tsRNAs. Depending on their length and the specific region in which endonucleases cleave tRNAs or pre-tRNAs, two primary forms of tsRNAs can be generated: (i) Cleavage of tRNA by ANG and Rny1 ribonucleases near or within the anticodon loop results in the production of 5’- or 3’- tiRNA halves with a length of 30–40 nucleotides. (ii) tRFs are sequences of 18–22 nucleotides generated when nucleases like Dicer or ANG, cleave tRNA at any location. tsRNAs: tRNA-derived small RNAs, ANG: Angiopoietin, tiRNAs: tRNA-derived stress-induced RNAs, tRFs: tRNA-derived fragments,

In addition to piRNAs, there are reports on the roles of tRNA-derived small RNAs (tsRNAs) and rRNAs in male infertility as well. tsRNAs are a novel family of regulatory RNAs generated through the cleavage of pre-tRNAs or tRNAs at specific sites by endonucleases. These molecules can be categorized into two subtypes based on their length and origin: tRNA-derived stress-induced RNAs (tiRNAs) and tRNA-derived fragments (tRFs) (Fig. 6) [161]. tsRNAs are involved in various biological activities, such as gene silencing, protein translation control, and reverse transcription regulation [162].

Hua et al. collected 87 human sperm samples from male partners of couples undergoing IVF and subsequently classified them into two groups based on the percentage of healthy embryos: high rate of good-quality embryos (H-GQE) and low rate of good-quality embryos (L-GQE). They identified 1899 tsRNAs within the sperm samples, wherein ten of these exhibited differential expression between the two groups. In the L-GQE group, five tsRNAs were upregulated (ProAGG-32, ProTGG-32, ProAGG-31, AsnATT-20, and ArgCCG-33), while the remaining ones showed downregulation (GlyGCC-30-1, GlyGCC-30-2, ThrTGT-38, ThrTGT-39, and GluTTC-23). They concluded that improper sperm tsRNA regulation might be a factor contributing to low sperm quality and aberrant early embryonic development [163]. In another study on the function of sperm tsRNAs in acquired metabolic illnesses that are passed down through generations, it was shown that injecting sperm tsRNA fractions from high-fat diet males into healthy zygotes can cause metabolic abnormalities in F1 offspring. It was concluded that sperm tsRNAs are paternal factors that may mediate the intergenerational transmission of a diet-induced metabolic diseases [164]. On the other hand, since metabolic disorders can affect semen quality and male fertility, these tsRNAs may also be important in infertility [165]. Bansal et al. found that ribosomal protein transcripts (5 S, 5.8 S, 18 S, 28 S, and 45 S) are differentially expressed in the sperm of asthenozoospermic and idiopathic infertile males compared to fertile individuals [166].

### Genomic imprinting

Mammals have been shown to exhibit parent-of-origin patterns of monoallelic gene expression, a process known as genomic imprinting. Over a hundred genes are regulated through this phenomenon, many of which play crucial roles in early developmental processes [167]. Variations in the parental genetic material initially occur within the germline as the two parental genomes become physically distinct. This process involves marking of discrete DNA regions through DNA methylation in one of the germlines [168]. DNA methylation patterns undergo dynamic alterations throughout spermatogenesis, playing pivotal roles in regulating gene expression and male fertility [169]. During DNA methylation reprogramming, PGCs experience a sequence of epigenetic changes. Initially, PGCs undergo demethylation as they expand and migrate. However, this demethylated state is transient, as the subsequent stages, particularly during spermatogenesis, involve a re-methylation process. Following fertilization, another phase of demethylation occurs before embryo implantation. Notably, imprinted genes, characterized by their distinctive and stable methylation patterns, manage to escape this demethylation wave [170]. Aberrant DNA methylation of imprinted genes has been proposed to be one of the possible factors in impaired spermatogenesis and male infertility [171]. While the precise etiology of the role of imprinting abnormalities in male reproductive impairments remains unknown, a list of identified imprinted genes implicated in male infertility is presented in Table 2. For instance, a study on boars revealed that while there was no difference in global methylation levels between fertile and infertile individuals, specific imprinted genes, such as MEG3, PEG10, DAZL, IGF2, and GNAS, were differentially methylated in infertile boars with low sperm quality [55]. Furthermore, experiments involving Dnmt3l knockout mice have shown that the absence of this enzyme can lead to disruptions in the methylation of imprinting genes, such as SNRPN and IGF2. These disruptions were associated with spermatocyte arrest and failure of meiosis in male mice [172, 173].

**Table 2:** List of imprinted genes associated with sperm-related infertility

Gene	Imprinted allele	Methylation status in abnormal condition	Condition	Reference
DIRAS3	Maternal	Aberrant DNA methylation	Infertile males vs. fertile donors	[214]
GNAS	Maternal	Aberrant DNA methylation	Infertile males vs. fertile donors	[214]
GNAS	?	Hypomethylated	Asthenozoospermia vs. normal controls	[215]
H19	Paternal	Hypomethylated	Oligozoospermia vs. Normozoospermia	[216]
H19	Paternal	Hypomethylated	[Obstructive] Azoospermia vs. fertile donors	[46]
H19	Paternal	Hypomethylated	A systematic review on infertile patients vs. fertile donors	[47]
H19	Paternal	Hypomethylated	Oligozoospermia vs. fertile donors	[51]
H19/IGF2-CTCF6	Paternal	Hypomethylated	Oligozoospermia vs. Normozoospermia	[53]
H19-ICR	Paternal	Hypomethylated	Oligoasthenozoospermia vs. Normozoospermia	[206]
H19-ICR	Paternal	Hypomethylated	Terato-asthenozoospermia vs. Normozoospermia	[206]
IGF2	Maternal	Hypermethylated	Mild asthenozoospermia vs. Normozoospermia	[217]
IGF2	Maternal	Hypermethylated	Severely impaired sperm DNA group (DFI > 30%) vs. low DFI group (DFI ≤ 30%)	[217]
IGF2	Maternal	Hypomethylated	DFI ≥ 25% vs. DFI < 25%	[218]
IGF2-DMR2	Paternal	Hypomethylated	Oligozoospermia vs. Normozoospermia	[53]
KCNQ1	Paternal	Hypomethylated	Mild asthenozoospermia vs. Normozoospermia	[217]
LIT1(KCNQ1OT1)	Maternal	Hypermethylated	Oligozoospermia vs. fertile donors	[51]
LIT1(KCNQ1OT1)	Maternal	Hypermethylated	Patients with abnormal sperm parameters vs. Normozoospermia	[219]
MEG3 (GTL2)	Paternal	Hypomethylated	Severely impaired sperm DNA group (DFI > 30%) vs. low DFI group (DFI ≤ 30%)	[217]
MEG3 (GTL2)	Paternal	Hypomethylated	Infertile males vs. fertile donors	[220]
MEG3/DLK1	Paternal	Hypomethylated	Oligozoospermia vs. Normozoospermia	[53]
MEST (PEG1)	Maternal	Hypomethylated	Mild asthenozoospermia vs. Normozoospermia	[217]
MEST (PEG1)	Maternal	Hypomethylated	Severely impaired sperm DNA group (DFI > 30%) vs. low DFI group (DFI ≤ 30%)	[217]
MEST (PEG1)	Maternal	Hypermethylated	Oligozoospermia vs. fertile donors	[51]
MEST (PEG1)	Maternal	Hypermethylated	Oligozoospermia vs. Normozoospermia	[49]
MEST (PEG1)	Maternal	Hypermethylated	Oligozoospermia vs. Normozoospermia	[216]
PEG3	Maternal	Hypomethylated	Severely impaired sperm DNA group (DFI > 30%) vs. low DFI group (DFI ≤ 30%)	[217]
PEG3	Maternal	Hypermethylated	Oligozoospermia vs. fertile donors	[51]
PLAGL1 (ZAC)	Maternal	Hypermethylated	Oligozoospermia vs. fertile donors	[51]
SNRPN	Maternal	Hypomethylated	Infertile males vs. fertile donors	[220]
SNRPN	Maternal	Hypermethylated	Infertile vs. fertile men	[205]
SNRPN	Maternal	Hypermethylated	Oligozoospermia vs. fertile donors	[51]
SNRPN-ICR	Maternal	Hypermethylated	Terato-asthenozoospermia vs. Normozoospermia	[206]
SNRPN-ICR	Maternal	Hypermethylated	Oligoasthenozoospermia vs. Normozoospermia	[206]
SNURF	Maternal	Hypermethylated	Oligozoospermia vs. Normozoospermia	[53]

DFI: DNA fragmentation index

### Environmental factors

Environmental factors, such as endocrine disrupting chemicals (EDCs) and lifestyle elements, have the potential to affect germline epigenetic markers, with sperm cells being the ultimate recipients of these changes [174].

ROS are free radicals from oxygen derivatives which can be produced internally or by external factors [175]. In a study conducted by Tunc et al., it was indicated that sperm from infertile men exhibited higher levels of seminal ROS and DNA fragmentation compared to fertile individuals. Additionally, there was a positive association between seminal ROS production and DNA fragmentation, whereas DNA methylation was negatively correlated with both factors. Overall, these findings suggest that oxidative stress-induced DNA damage may contribute to abnormal DNA methylation patterns in sperm of infertile patients [176].

Multiple studies have linked EDCs to disturbances in spermatogenesis and male infertility via epigenetic pathways. Exposure to zearalenone during puberty has been shown to disrupt meiosis and signaling pathways during spermatogenesis, resulting in reduced semen quality in mice [177]. Bisphenol A (BPA) which is an industrial chemical found in various products like food cans, milk containers, baby bottles, and dental sealants, has been repeatedly reported to affect sperm epigenetics [178]. It has been shown to increase oxidative stress in the testes and epididymal sperm of rats, leading to reduced sperm chromatin condensation and disrupted sperm nuclear structure [179]. Another study found that methylation levels of LINE-1 in sperm was significantly lower in workers exposed to BPA compared to control group, potentially resulting in increased genetic instability [180]. Another EDC is Atrazine, which is an herbicide commonly used in farming that often pollutes water sources [181]. It was shown that brief exposure to this substance during gestation can lead to differential DNA methylation patterns in the sperm of F1-F3 rat generations [182].

Cigarette smoking is a known risk factor for male infertility due to its high levels of free radicals, which can trigger the generation of ROS in the body [183]. It has the potential to negatively affect epigenetic signatures of sperm [20]. Previous studies have revealed that smoking can change the human sperm histone-to-protamine ratio [184, 185] and non-coding RNA expression levels [185]. Hao et al., reported that smoking can be an important factor in male infertility by triggering hypomethylation of H19-ICR in oligozoospermia and hypermethylation of SNRPN-ICR in asthenozoospermia and teratozoospermia patients [186].

Accumulating evidence suggests that dietary elements can alter epigenetic markers [187]. According to various animal studies, a high-fat diet is accompanied by the appearance of differentially methylated sites and an increase in the expression of sperm miRNA let-7c [188]. Moreover, it has been linked to increased levels of acetylated H3K9, which associates with decreased SIRT6 protein levels and DNA damage in the sperm [189]. Sperm from mice fed a low-protein diet exhibited increased H3K27me3 levels in certain genes. This can lead to histone retention [190], a phenomenon previously associated with male infertility [191]. Oakes et al., found that the anticancer drug 5-aza-20-deoxycytidine inhibits de novo DNA methylation in male germ cells, impacting genes which are usually methylated during sperm development resulting in changes in sperm structure, reduced motility, lower fertility, and embryo viability [192].

### Concluding remarks

Identifying the primary molecular factors and underlying contributors of male infertility remains a significant challenge in reproductive medicine [193]. This review emphasizes the involvement of various sperm epigenetic factors in regulating multiple layers of genes that may be crucial for male fertility. Acknowledging epigenetics along with other factors such as genetic modifications, can enrich our understanding of the etiology of male infertility, offering insights beyond what genetic modifications alone provide [194]. Since epigenetic changes are reversible, studying them in different diseases raises the possibility of designing certain drugs for that condition [195]. While establishing definitive causal connections between epigenetic markers in sperm and fertility has proven challenging, a substantial body of evidence suggests associations between them, along with adverse pregnancy outcomes [196]. It is becoming increasingly apparent that many epigenetic modifications observed in infertility are interrelated. Deviations in methylation levels are associated with an imbalance between histones and PRMs. The cumulative effects of these epigenetic changes not only plays role in male fertility but also affect the formation and development of the embryo and the health of the offspring. When studying epigenetic changes in sperm, it is important to consider technical and biological limitations. To ensure accurate results, it is crucial to remove somatic cells since sperm has distinct epigenetic fingerprints compared to other types of cells. Research on male infertility will benefit substantially from larger sample sizes and integration of multiple epigenetic layers. Given the considerable cellular diversity exhibited by sperm, as well as the documented epigenetic heterogeneity in this cell population, it will be of significant importance for future studies to employ high-throughput single-cell epigenetic methods. In conclusion, the current review reveals that sperm serves a role beyond merely transporting its haploid genome to the oocyte. The presence of DNA methylation, chromatin modification, and sperm-born ncRNAs forms a distinctive epigenetic environment in sperm that can be related to male infertility. However, it is important to acknowledge the complexity of this relationship. While many studies have identified epigenetic abnormalities in infertile males, it is crucial to recognize that these abnormalities may not always be the primary cause. Instead, they could represent a contributing pathway to infertility, influenced by factors such as environmental exposures. Therefore, although epigenetic changes are commonly observed in male infertility cases, they may not always be the sole determinant and further research is needed to definitively establish the role of epigenetics in male infertility.

## Data Availability

No datasets were generated or analysed during the current study.

## Abbreviations

ARTs: Assisted reproductive techniques
cP2: cleaved PRM2
DNMTs: DNA methyltransferases
ERs: Estrogen receptors
Hypac-H4: Histone H4 hyperacetylation
ICR: Imprinting control region
ICSI: Intracytoplasmic sperm injection
IVF: In-vitro fertilization
lncRNAs: long non-coding RNAs
miRNAs: microRNAs
mP2: mature PRM2
ncRNAs: non-coding RNAs
piRNAs: piwi-interacting RNAs
PGCs: Primordial germ cells
PRM: Protamine
PSA: Prostate-specific antigen
ROS: Reactive oxygen species
SDF: Sperm DNA fragmentation
siRNAs: small interfering RNAs
snoRNAs: small nucleolar RNAs
tiRNAs: tRNA-derived stress-induced RNAs
tRFs: tRNA-derived fragments
tRNA: transfer RNA
tsRNAs: tRNA-derived small RNAs
UTR: Untranslated region
WHO: World health organization
